# Predicting disease progression in advanced non-small cell lung cancer with circulating neutrophil-derived and platelet-derived microparticles

**DOI:** 10.1186/s12885-021-08628-4

**Published:** 2021-08-21

**Authors:** Tingting Liu, Jiang Wang, Tao Li, Pengfei Cui, Baicun Hou, Chunxiao Zhuang, Ge Wei, Sujie Zhang, Hongxia Li, Yi Hu

**Affiliations:** 1 Medical School of Chinese PLA, Chinese PLA General Hospital, Beijing, 100853 China; 2 Department of Medical Oncology, The First Medical Center, Chinese PLA General Hospital, Beijing, 100853 China; 3 Department of Pulmonary and Critical Care Medicine, The Second Medical Center, National Clinical Research Center for Geriatric Diseases, Chinese PLA General Hospital, Beijing, 100853 China; 4 Centre of Pulmonary and Critical Care Medicine, Chinese PLA General Hospital, Beijing, 100853 China; 5 People Liberation Army Haidian District 17th Retired Cadres Rest Home, Beijing, 100143 PR China

**Keywords:** Neutrophil-derived microparticles, Platelet-derived microparticles, Non-small cell lung cancer, Progressive disease, Predictive model

## Abstract

**Background:**

Microparticles (MPs) are extracellular vesicles that are associated with cancer development and progression. Advanced non-small cell lung cancer (NSCLC) still shows disease progression after multiple lines of treatment. Therefore, the objective of this study was to explore the correlation between circulating MPs and disease progression in advanced NSCLC, and to find a new method for concise and rapid determination of disease progression.

**Methods:**

Patients with advanced NSCLC admitted to hospital between October 2019 and October 2020 were included and divided into objective remission (OR) and progressive disease (PD) groups. The morphology of MPs was observed using transmission electron microscopy. The circulating total MPs, neutrophil MPs (NMPs), and platelet MPs (PMPs) before and after treatment were detected by flow cytometry, and a predictive model for disease progression in advanced NSCLC was developed.

**Results:**

Eighty-six patients were included; 60 in the OR group and 26 in the PD group. There was no significant difference in total MPs, NMPs, or PMPs at baseline between the two groups. After treatment, total MPs, NMPs, and PMPs were significantly higher in the PD than those in the OR group. Multivariate regression analysis showed that post-treatment NMPs≥160 events/μL(OR,3.748;95%CI,1.147–12.253,*p* = 0.029), PMPs≥80 events/μL(OR,10.968;95%CI,2.973–40.462,*p* < 0.0001) and neutrophil/lymphocyte ratio (NLR) ≥3.3 (OR,4.929;95%CI,1.483–16.375,*p* = 0.009) were independently associated with progression of advanced NSCLC. Post-treatment NMPs and PMPs combined with NLR were used to build a predictive model for progression of advanced NSCLC. The area under the curve was 0.825 (95%CI,0.715–0.934, *p* < 0.0001), optimal cut-off value was 16, sensitivity was 80.8%, and specificity was 88.3%.

**Conclusion:**

NMPs and PMPs are associated with progression of advanced NSCLC. The predictive model for progression of advanced NSCLC, established combining NMPs, PMPs, and NLR, can screen out 80.8% of patients with PD. This is helpful for real-time accurate, concise and rapid assessment of disease progression and timely adjustment of drug therapy.

**Trial registration:**

Chinese Clinical Trial Registry, ChiCTR1800020223. Registered 20 December 2018, http://www.chictr.org.cn/index.aspx.

## Introduction

Lung cancer is a common malignancy, accounting for 11.6% of total cancer incidence and 18.4% of cancer deaths, and remains the leading cause of cancer death worldwide [1, 2]. Non-small cell lung cancer (NSCLC) accounts for 85–90% of lung cancers, and radiotherapy and chemotherapy are still important for treatment of advanced NSCLC, but the 5-year survival rate is < 15% [3]. In recent years, targeted therapy and immunotherapy have been widely used for advanced NSCLC [4–6], but disease progression still occurs in advanced NSCLC after multiple lines of treatment. Therefore, exploration of new mechanisms that promote cancer progression, and rapid and concise assessment of disease progression to guide and adjustment of drug therapy are important to prolong patient survival.

Microparticles (MPs) are submicron vesicles generated from various types of cells and were initially considered functionless cellular products. However, an increasing number of studies have revealed that MPs are not metabolic waste products of cells and are involved in coagulation, cell signaling, vascular injury, and stabilization of the internal environment [7]. In 2005, MPs were defined as cell-derived, extracellular vesicles of 0.1–1 μm diameter that lack a nucleus and the ability to synthesize proteins. They carry parent-cell-derived proteins (e.g., signaling proteins, skeletal proteins, etc.), nucleic acids (e.g., miRNA, mRNA, DNA), and lipids, and have high level of membrane phosphatidylserine [8]. MPs are elevated in a variety of diseases, including acute coronary syndromes, septic shock, and rheumatic immune system diseases [7, 8]. In cancer, MPs are derived from platelets and endothelial, inflammatory and tumor cells, and are associated with cancer staging, thrombosis, angiogenesis, proliferation and metastasis [9–12].

The peripheral blood neutrophil/lymphocyte ratio (NLR) has been shown to correlate with the prognosis and progression of several cancers [13–15]. Tumor-associated neutrophils (TAMs) promote tumor progression by producing reactive oxygen and reactive nitrogen species, releasing oncostatin M, and remodeling the tumor extracellular matrix (ECM) [16, 17]. Neutrophil MPs (NMPs) originate from neutrophils, and we speculate that NMPs contain neutrophil-associated signaling molecules that promote cancer progression. As the most-abundant circulating MPs [18], platelet MPs (PMPs) are closely associated with high invasiveness, metastasis and poor prognosis of cancer [19, 20]. Our previous studies have found that PMPs can independently predict the efficacy of immunotherapy [21]. Therefore, in this study, we investigated the correlation between circulating NMPs and PMPs and progression of advanced NSCLC, and established a model for predicting disease progression by combining laboratory indices, to provide clinicians with a new method for timely identification of disease progression.

## Materials and methods

### Patients

Patients with advanced NSCLC between October 2019 and October 2020 at our hospital were enrolled. Inclusion criteria: patients older than 18 years old; advanced non-small cell lung cancer (stage III or IV). Exclusion criteria: other malignant tumours; severe organ dysfunction, such as chronic renal insufficiency (stage III and above), liver cirrhosis (Child-Pugh classification C and above); acute infection; autoimmune diseases; and current radiotherapy.

### Blood sampling

Before and after 20–28 weeks of treatment, the routine blood, lactate dehydrogenase, and tumor markers were routinely examined, and 3 ml of venous blood from the elbow was extracted for the detection of MPs. To early detect of disease progression and avoid the effects of therapeutic drugs on MPs, blood samples were collected before use of therapeutic drugs on the same day and at least 21 days after the last medication. MPs isolation by differential centrifugation was performed: 3 mL of whole blood was centrifuged (2000 g) for 10 min to obtain platelet-poor plasma (PPP); then, PPP was ultracentrifuged (13,000 g) for 3 min to collect platelet-free plasma (PFP), which was stored in the refrigerator at − 80 °C.

### Microparticle analysis

#### Observation of MPs morphology by transmission electron microscopy

MPs (20 μl) from patients were placed on copper mesh and incubated for 5 min. We removed the remaining liquid with filter paper, and added 20 μl phosphotungstic acid. After standing for 10 min, any remaining liquid was removed with filter paper, and the sample was observed by transmission electron microscopy.

#### Detection of MPs number by flow cytometry

**Fig. 1:**
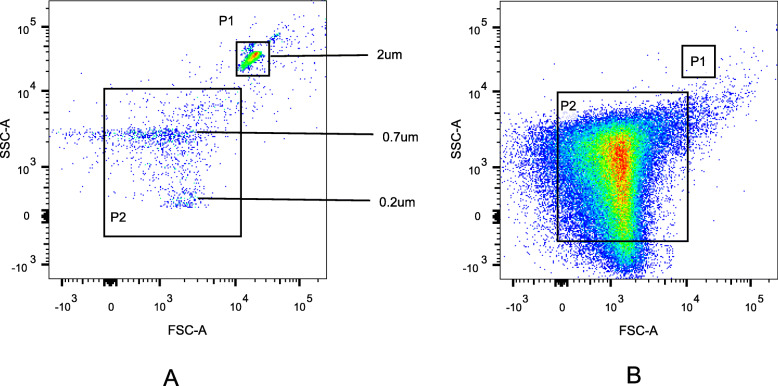
The setting of gates in the flow cytometer was shown in Fig. 1. The gates in A represented the positions of particles with diameters of 2 μm, 0.7 μm, 0.2 μm respectively, and P2 was the position of particles with diameters of 0.1–1 μm, that was, the position of the MPs. B showed the flow diagram of MPs on the tester, and most MPs were in the P2 gate

PFP was dissolved at room temperature and then ultracentrifuged (4 °C, 21,000 g) for 60 min, and 100 μL of the bottom fraction was obtained as MPs. The number of MPs was detected by FACSCanto™ II flow cytometer (BD Bioscience, San Jose, CA, USA). The immunofluorescent monoclonal antibodies used were as follows (BioLegend, San Diego, CA, USA): Annexin V labeled by PE, CD66b and CD41 labeled by FITC. CD66b+/Annexin V+ represents NMPs, CD41+/Annexin V+ represents PMPs. Moreover, 25 μL of MPs, 1 μL Annexin V and 100 μL Annexin V binding buffer were added into two Eppendorf tubes A and B, and then incubated for 20 min at room temperature, avoiding light. Then, 1 μL CD66b-FITC were added into tube A, and 1 μL CD41-FITC were added into tube B and incubated for 20 min at room temperature, avoiding light. Then, both tubes A and B were washed with phosphate-buffered saline (PBS), transferred to BD absolute counting tubes, and tested on the machine. Furthermore, 0.2 μm, 0.7 μm, and 2 μm standard microspheres (invitrogen, carlsbad, CA, USA) were used to set the gate and determine the location of 0.1–1.0-μm MPs (Fig. 1). To avoid the noise caused by dust and crystallization, the buffer and PBS were filtered through a 0.22-μm filter. MPs were counted in events/μL.

### Therapeutic evaluation

Before and after 20–28 weeks of treatment, chest and abdominal computed tomography (CT) and head magnetic resonance imaging (MRI) were performed to determine the stage of cancer and evaluate the therapeutic effect. The efficacy of non-immunotreated patients was evaluated by the 2009 Response Evaluation Criteria in solid tumor (RECIST 1. 1) [22], while the efficacy of immunotreated patients was evaluated by the 2017 Immune-related Response Evaluation Criteria in Solid Tumours (iRECIST) [23]. The patients were divided into objective remission (OR) and progressive disease (PD) groups. The OR group included patients with complete remission (CR), partial remission (PR) or stable disease (SD). PD was defined as a change in the longest diameter of the tumour on CT examination ≥20% or the occurrence of new metastatic lesions.

### Statistical analysis

Count data was compared by chi-square test or Fisher’s exact probability test. Quantitative data of normal distributions represented by x ± s were analyzed by t-tests. Quantitative data of nonnormal distributions presented as the medians and interquartile ranges (IQRs) were analyzed by Mann-Whitney U test. Univariate analysis and binary multivariate logistic regression analysis (Forward: LR) were used to find the independent influencing factors. The scores of independent factors were evaluated according to the OR value, that is, Score≈OR, and then the total score was calculated by adding the scores of each factor [24]. We also established a prediction model for disease progression. The sensitivity and specificity of the prediction model were tested by receiver operating characteristic (ROC) curve. *P* < 0.05 were statistically signifificant.

## Result

### MPs morphology

**Fig. 2:**
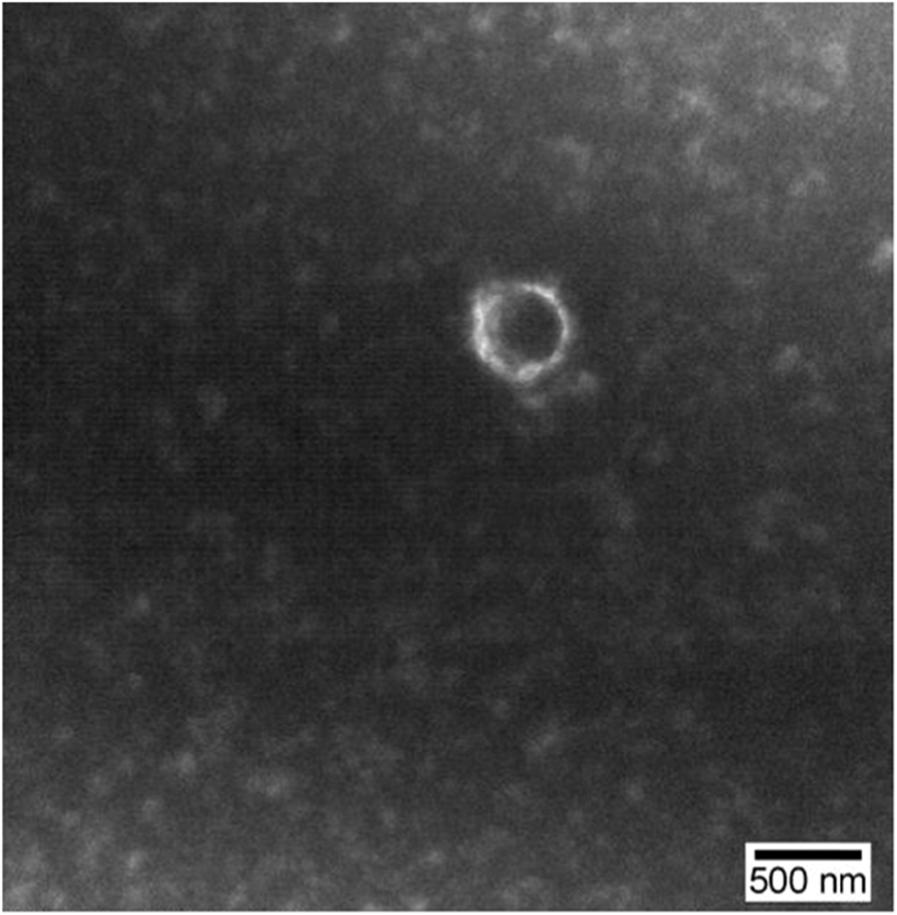
The morphology of microparticles (MPs) was observed using transmission electron microscopy. MPs had spherical structures under electron microscopy, with diameters ranging from 100 to 1000 nm, heterogeneity in size, and typical lipid bilayer structure

MPs have spherical structures with diameters ranging from 100 to 1000 nm, heterogeneity in size, and typical lipid bilayer structure (Fig. 2).

### Comparison of clinical characteristics and baseline MPs between 86 cases of NSCLC in the OR and PD groups

There were 86 patients, with 60 in the OR group and 26 in the PD group. There were no significant differences between the two groups in terms of gender, age, smoking history, pathological staging, tumor stage, metastasis, Karnofsky’s index of performance status (KPS) score, number of treatment lines and therapeutic intervention (*p* > 0.05, Table 1). Chemotherapy regiments were mainly platinum-containing dual-agent regiments, immunotherapy included pembrolizumab and nivolumab, and target therapy included gefitinib, icotinib, erlotinib and osimertinib. There was no significant difference in baseline laboratory indices, total MPs, NMPs and PMPs between the two groups (*p* > 0.05, Table 1).

**Table 1:** Baseline variables and micropaticles in OR group and PD group of 86 cases of NSCLC

Variables	OR (*n* = 60)	PD (*n* = 26)	P
Male, gender, n(%)	45 (75.0)	19 (73.1)	0.851^*^
Age, years, x ± s	61.8 ± 10.7	62.6 ± 11.1	0.760^§^
Current or former smokers, n(%)	31 (51.7)	17 (65.4)	0.239^*^
Histology, n(%)			0.297^*^
Adenocarcinoma	25 (41.7)	14 (53.8)	
Squamous carcinoma	35 (58.3)	12 (46.2)	
Disease stage, n(%)			0.274^*^
Stage III	26 (43.3)	8 (30.8)	
Stage IV	34 (56.7)	18 (69.2)	
Lines of therapy, n (%)			0.759^*^
<2	21 (35.0)	10 (38.5)	
≥ 2	39 (65.0)	16 (61.5)	
Therapeutic intervention, n(%)			0.343^*^
Chemotherapy+Immunotherapy	31 (51.7)	10 (38.5)	
Chemotherapy	21 (35.0)	9 (34.6)	
Immunotherapy	5 (8.3)	3 (11.5)	
Target therapy	3 (5.0)	4 (15.4)	
No. of metastatic organs, n(%)			1.000^*^
<2	30 (50.0)	13 (50.0)	
≥ 2	30 (50.0)	13 (50.0)	
No. of metastatic lymph nodes, n(%)			0.773^*^
<2	18 (30.0)	7 (26.9)	
≥ 2	42 (70.0)	19 (73.1)	
KPS score, n(%)			0.733^*^
90	54 (90.0)	22 (84.6)	
80	4 (6.7)	2 (7.7)	
70	2 (3.3)	2 (7.7)	
Laboratory indices at baseline, median(IQR)			
Neutrophils, ×10^9^/L	3.61 [2.70,4.50]	3.62 [2.87,4.63]	0.929^*#*^
Platelets, ×10^9^/L	205 [154,258]	200 [144,265]	0.914^*#*^
NLR	3.32 [2.11,4.60]	3.27 [1.75,4.56]	0.803^*#*^
LDH, U/L	191 [160,229]	188 [164,237]	0.566^*#*^
CEA, ng/ml	5.10 [1.94,32.71]	5.23 [1.81,36.86]	0.925^*#*^
CYFRA 21-1, ng/ml	5.14 [3.33,7.93]	5.50 [3.33,8.98]	0.413^*#*^
SCC, ng/ml	1.10 [0.85,1.63]	1.08 [0.82,1.75]	0.745^*#*^
Micropaticals at baseline, events/μL, median(IQR)			
total MPs	2632 [1942,3600]	2586 [1311,3490]	0.771^*#*^
NMPs	164 [118,317]	217 [121,267]	0.836^*#*^
PMPs	205 [154,258]	174 [143,211]	0.951^*#*^

*KPS* Karnofsky’s index of performance status, *NLR* neutrophil-lymphocyte ratio, *LDH* lactate dehydrogenase, *CEA* carcino-embryonic antigen, *CYFRA-211* Cytokeratin 19 fragment, *SCC* Squamous cell carcinoma antigen, *MPs* microparticals, *NMPs* neutrophil-derived microparticals, *PMPs* platelet-derived microparticles

^*^Chi-square test

^§^t test

^#^Mann-Whitney U test

### Comparison of laboratory indices and MPs after treatment between 86 cases of NSCLC in the OR and PD groups

After treatment, there was no significant difference in neutrophils, platelets and squamous cell carcinoma antigen between the two groups (*p* > 0.05, Table 2). NLR, lactate dehydrogenase (LDH), carcinoembryonic antigen, and cytokeratin 19 fragment (CYFRA 21-1) were significantly higher in the PD group than those in the OR group (*p* < 0.05, Table 2).

**Table 2:** Micropaticles and laboratory data after treatment in OR group and PD group

Variables	OR (*n* = 60)	PD (*n* = 26)	P
Neutrophils, ×10^9^/L, median(IQR)	3.62 [2.70,4.50]	4.30 [2.93,5.35]	0.232^*#*^
Platelets, ×10^9^/L, median(IQR)	205 [154,258]	196 [160,281]	0.807^*#*^
NLR, median(IQR)	2.43 [1.62,3.23]	3.37 [1.75,7.04]	0.029^*#*^
LDH, U/L, median(IQR)	166 [142,200]	188 [169,240]	0.004^*#*^
CEA, ng/ml, median(IQR)	2.85 [1.95,12.76]	8.72 [3.29,50.13]	0.026^*#*^
CYFRA-211, ng/ml, median(IQR)	3.15 [2.14,4.08]	4.54 [3.30,8.11]	< 0.0001^*#*^
SCC, ng/ml, median(IQR)	1.10 [0.93,1.58]	1.05 [0.68,1.93]	0.491^*#*^
Micropaticals, events/μL, median(IQR)			
total MPs	1975 [1090,3120]	3590 [1492,4405]	0.006^*#*^
NMPs	118 [66,229]	248 [116,338]	0.002^*#*^
PMPs	56 [31,109]	157 [93,282]	<0.0001^*#*^

*NLR* neutrophil-lymphocyte ratio, *LDH* lactate dehydrogenase, *CEA* carcino-embryonic antigen, *CYFRA-211* Cytokeratin 19 fragment, *SCC* Squamous cell carcinoma antigen, *MPs* microparticals, *NMPs* neutrophil-derived microparticals, *PMPs* platelet-derived microparticles

^#^Mann-Whitney U test

**Fig. 3:**
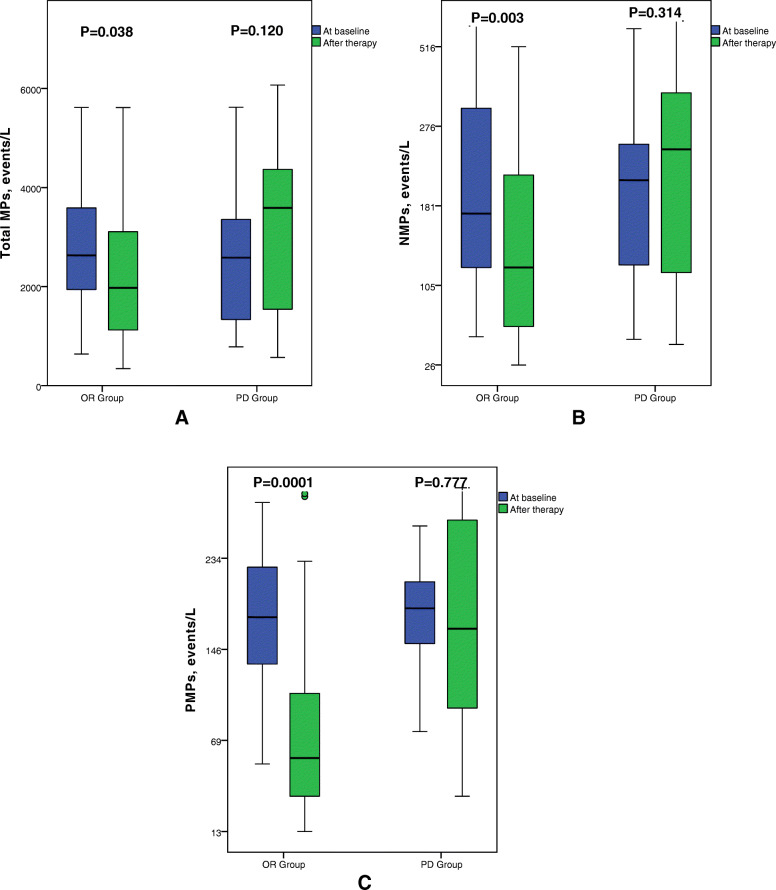
Box plots for circulating microparticles levels at baseline and after treatment in the OR and PD groups. Top and bottom of box represented 75th and 25th percentile, respectively; middle bar in box represented the median value; Top and bottom lines extending from box represented maximum and minimum obtained values, respectively. ° represented mild outliers. In PD group, total MPs, NMPs and PMPs did not change significantly after treatment (*p* > 0.05). In OR group, total MPs, NMPs and PMPs were decreased significantly after treatment (*p* < 0.05). A showed total MPs, B showed NMPs, C showed PMPs

**Fig. 4:**
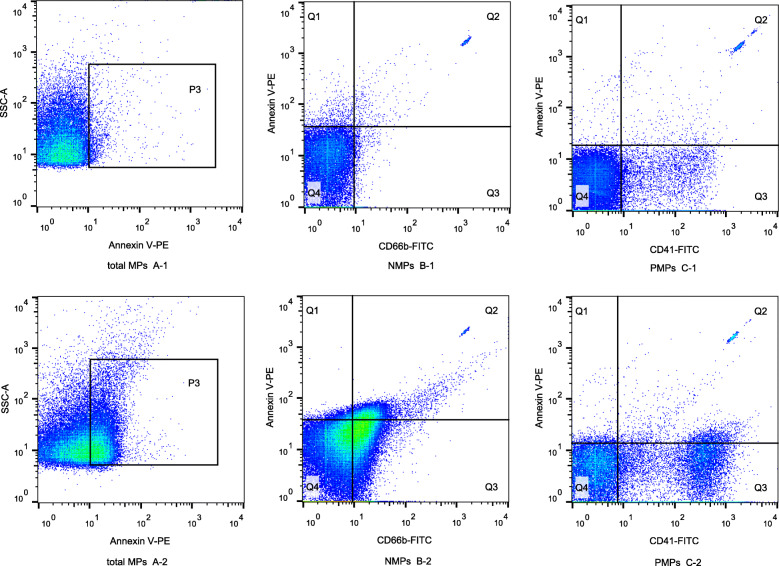
Flow chart of MPs in the OR and PD groups. A-1 and A-2 showed total MPs in the OR and PD groups after treatment, and total MPs were significantly higher in the PD group than those in the OR group. B-1 and B-2 showed NMPs in the OR and PD groups after treatment, and NMPs were significantly higher in the PD group than those in the OR group. C-1 and C-2 showed PMPs in the OR and PD groups after treatment, and PMPs were significantly higher in the PD group than those in the OR group

In the OR group, the circulating total MPs, NMPs and PMPs after treatment were 1975[1090,3120]events/μL, 118[66,229]events/μL, and 56[31,109]events/μL, respectively. The total MPs, NMPs and PMPs in the OR group were significantly lower than those at baseline (*p* < 0.05, Fig. 3). In the PD group, the total MPs, NMPs and PMPs after treatment were 3590[1492,4405]events/μL, 248[116,338] events/μL, and 157[93,282]events/μL, respectively. There was no significant difference in total MPs, NMPs or PMPs in the PD group compared with the baseline levels (*p* > 0.05, Fig. 3). Total MPs, NMPs, and PMPs were significantly higher in the PD group than those in the OR group after treatment (*p* < 0.005, Table 2, Figs. 3 and 4).

### Multivariate logistic regression analysis for predicting the progression of advanced NSCLC

Factors with *P* < 0.1 in the univariate analysis were included in the multivariate regression analysis. The results showed that NMPs≥160events/μL(OR,3.748;95%CI,1.147–12.253,*p* = 0.029), PMPs≥80events/μL(OR,10.968;95%CI,2.973–40.462, *p* < 0.0001), and NLR ≥ 3.3 (OR,4.929;95%CI,1.483–16.375,*p* = 0.009) were independently associated with progression of advanced NSCLC. On the basis of the multivariate regression analysis, significant factors were scored and a predictive model for progression of advanced NSCLC was established (Table 3), for which a prediction score > 16 was considered high risk and ≤ 16 was low risk.

**Table 3:** Multivariate analyses of factors related with PD in advanced NSCLC

Variables	β	S.E.	Wald	df	Sig.	OR	95% CI for OR		Score
							Lower	Upper	
NMPs, ≥160 events/μL	1.321	0.604	4.779	1	0.029	3.748	1.147	12.253	4
PMPs, ≥80 events/μL	2.395	0.666	12.930	1	<0.0001	10.968	2.973	40.462	11
NLR, ≥3.3	1.595	0.613	6.779	1	0.009	4.929	1.483	16.375	5
Constant	−3.607	0.767	22.123	1	0.000	0.027			

*NMPs* neutrophil-derived microparticals, *PMPs* platelet-derived microparticles, *NLR* neutrophil-lymphocyte ratio, *CYFRA-211* Cytokeratin 19 fragment

### ROC curve to assess the efficacy of the predictive model

**Fig. 5:**
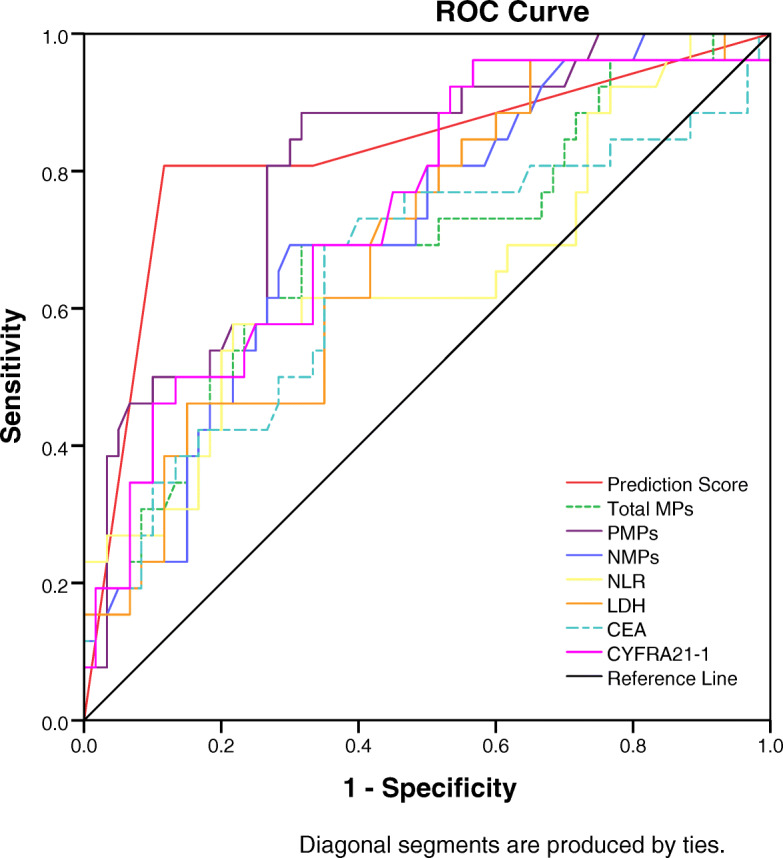
Receiver operating characteristic curves (ROC) displayed Prediction Score and other factors for predicting progression in advanced NSCLC. Area under the curve of Prediction Score was 0.825(95%CI,0.715–0.934, *p* < 0.0001)

The ROC curve for predicting progression of advanced NSCLC by prediction score and other related factors was plotted, and showed that the area under the curve for prediction score evaluation for progression of advanced NSCLC was 0.825(95%CI,0.715–0.934, *P* < 0.0001), with an optimal cut-off value of 16. The sensitivity was 80.8%, specificity was 88.3%, positive predictive value was 75.0%, negative predictive value was 91.4%, and Youden index was 0.691, which were significantly superior to other factors associated with cancer progression (Table 4, Fig. 5).

**Table 4:** Comparison of Prediction Score and other indicators in predicting the progression of advanced NSCLC

Variables	Cutoff	AUC 95% CI	Sensitivity	Specificity	PPV	NPV	P
Prediction Score	16	0.825 (0.715,0.934)	80.8%	88.3%	75.0%	91.4%	< 0.0001
total MPs, events/μL	2800	0.686 (0.559,0.813)	69.2%	68.3%	48.6%	83.7%	0.006
NMPs, events/μL	160	0.712 (0.598,0.827)	69.2%	65.0%	46.2%	83.0%	0.002
PMPs, events/μL	80	0.804 (0.706,0.903)	84.6%	68.3%	53.7%	91.1%	< 0.0001
NLR	3.3	0.648 (0.513,0.784)	57.7%	78.3%	53.6%	81.0%	0.029
CEA, ng/ml	5.0	0.652 (0.515,0.788)	69.2%	65.0%	46.2%	83.0%	0.026
CYFRA21-1, ng/ml	2.5	0.741 (0.628,0.855)	96.2%	43.3%	42.4%	96.3%	0.058
LDH, U/L	210	0.694 (0.576,0.812)	46.2%	85.0%	57.1%	78.5%	0.004

*PPV* Positive predictive value, *NPV* Negative predictive value, *MPs* microparticals, *NMPs* neutrophil-derived microparticals, *PMPs* platelet-derived microparticles, *CEA* carcino-embryonic antigen, *CYFRA-211* Cytokeratin 19 fragment, *NLR* neutrophil-lymphocyte ratio, *LDH* lactate dehydrogenase, *CEA* carcino-embryonic antigen

## Discussion

Patients with advanced NSCLC were included in this study. There was no difference in the circulating total MPs, NMPs, or PMPs before treatment. Since patients were in disease progression stage before treatment, the levels of circulating MPs were high in both groups. While after multiple cycles of treatment, circulating total MPs, NMPs, and PMPs were maintained at high baseline levels in patients with disease progression. In contrast, post-treatment circulating total MPs, NMPs, and PMPs were significantly lower compared with baseline levels in patients with stable disease. These levels were also significantly lower than those in patients with progressive disease, suggesting that MPs might promote cancer progression, which is consistent with the results of Kanazawa [25] and Najjar [26]. On the basis of circulating NMPs, PMPs, and NLR, we developed a predictive model for disease progression in advanced NSCLC, which suggests a high risk of disease progression when the prediction score is > 16. This provides a new method for judging the effect of drug therapy and timely detection of disease progression.

NMPs are significantly elevated in advanced NSCLC when disease progression occurs, suggesting that NMPs may promote cancer progression; however, the relevant mechanisms are unclear. We suggest that the promotion of cancer progression by NMPs might be related to the proinflammatory effects of NMPs. NMPs can activate JNK1 signaling in endothelial cells, induce the release of IL-6 and MCP-1 [27], promote IL-6 and IL-8 production by endothelial cells, and increase expression of adhesion molecules [28]. NMPs can also contribute to increased ICAM-1 expression in endothelial cells and increased reactive oxygen species production [29]; all of which lead to increased systemic inflammation and may promote cancer progression.

PMPs are strongly associated with high cancer aggressiveness, metastasis and poor prognosis [30, 31]. Helley et al. [32] found that PMPs promoted prostate cancer metastasis and led to poor prognosis. Kim [33] found that PMPs were significantly elevated in patients with advanced gastric cancer and were predictive of distant metastasis. Our previous study found that PMPs were significantly elevated in patients with advanced NSCLC and could independently predict immunotherapeutic efficacy [21]. In the present study, we also found that PMPs were associated with disease progression in advanced NSCLC, regardless of the treatment received. Circulating PMPs ≥80 events/μL indicates a poor therapeutic effect, and active imaging examination is required to determine whether disease progression is occurring.

Assessment standards for the treatment efficacy of solid tumors recommend the use of radiological imaging. However, efficacy is comprehensively evaluated every 6–12 weeks, which does not allow timely detection of disease progression, and identification of pseudo-progression during immunotherapy is difficult with imaging [22]. Significant elevations in tumor markers, NLR and LDH suggest poor cancer treatment response, but they have a lag in detection and low specificity. We found that circulating NMPs and PMPs were independently associated with disease progression in advanced NSCLC, suggesting that NMPs and PMPs may be novel mechanisms for promotion of tumor progression. A model using NMPs and PMPs combined with NLR for prediction of disease progression showed a sensitivity of 80.8% and specificity of 88.3%. When the prediction score indicates high risk, we can screen out 80.8% of patients with disease progression, and we need to actively improve imaging examination to determine disease progression and adjust drug therapy for these patients. When the prediction score for a patient is low, the patient has a 91.4% probability of stable disease, which eliminates the need for repeated imaging, avoids radiation toxicity, and reduces the economic burden on patients and hospitals.

According to our literature search, this study was the first to analyze the relationship between circulating NMPs and disease progression in advanced NSCLC, and multivariate regression analysis showed that NMPs and PMPs combined with NLR were able to predict disease progression. This study had some shortcomings: first, it was a single-center study with a small sample size; and second, the mechanism by which MPs promoted cancer progression was not further explored.

## Conclusion

We found that circulating NMPs and PMPs independently predicted disease progression in advanced NSCLC, and combination with NLR to build a predictive model for disease progression effectively screened 80.8% of patients with disease progression. We reveal that NMPs and PMPs may be new factors that promote cancer progression, and provide a new method for concise, real-time dynamic assessment of advanced NSCLC disease progression. This could help to adjust drug therapy and improve prognosis in a timely manner, and reduce the financial burden on patients.

## Data Availability

The data used and/or analyzed during the current study are available from the corresponding author on reasonable request.

## References

[1] Bray F, Ferlay J, Soerjomataram I, Siegel RL, Torre LA, Jemal A (2018). Global cancer statistics 2018: GLOBOCAN estimates of incidence and mortality worldwide for 36 cancers in 185 countries. CA Cancer J Clin.

[2] Mattiuzzi C, Lippi G (2019). Current Cancer epidemiology. J Epidemiol Glob Health.

[3] Du L, Morgensztern D (2015). Chemotherapy for advanced-stage non-small cell lung Cancer. Cancer J.

[4] Singh SS, Dahal A, Shrestha L, Jois SD (2020). Genotype driven therapy for non-small cell lung Cancer: resistance, Pan inhibitors and immunotherapy. Curr Med Chem.

[5] Tan AC (2020). Targeting the PI3K/Akt/mTOR pathway in non-small cell lung cancer (NSCLC). Thorac Cancer.

[6] Doroshow DB, Herbst RS (2018). Treatment of advanced non-small cell lung Cancer in 2018. JAMA Oncol.

[7] Mackman N (2009). On the trail of microparticles. Circ Res.

[8] Hargett LA, Bauer NN (2013). On the origin of microparticles: from "platelet dust" to mediators of intercellular communication. Pulm Circ.

[9] Litwińska Z, Łuczkowska K, Machaliński B (2019). Extracellular vesicles in hematological malignancies. Leuk Lymphoma.

[10] Rousseau A, Van Dreden P, Khaterchi A (2017). Procoagulant microparticles derived from cancer cells have determinant role in the hypercoagulable state associated with cancer. Int J Oncol.

[11] Arderiu G, Peña E, Badimon L (2015). Angiogenic microvascular endothelial cells release microparticles rich in tissue factor that promotes postischemic collateral vessel formation. Arterioscler Thromb Vasc Biol.

[12] Wu K, Xing F, Wu SY, Watabe K (2017). Extracellular vesicles as emerging targets in cancer: recent development from bench to bedside. Biochim Biophys Acta Rev Cancer.

[13] Kim TG, Park W, Kim H, Choi DH, Park HC, Kim SH, Cho YB, Yun SH, Kim HC, Lee WY, Lee J, Kang KM (2019). Baseline neutrophil-lymphocyte ratio and platelet-lymphocyte ratio in rectal cancer patients following neoadjuvant chemoradiotherapy. Tumori..

[14] Pirozzolo G, Gisbertz SS, Castoro C, van Berge Henegouwen MI, Scarpa M (2019). Neutrophil-to-lymphocyte ratio as prognostic marker in esophageal cancer: a systematic review and meta-analysis. J Thorac Dis.

[15] Nakaya A, Kurata T, Yoshioka H, Takeyasu Y, Niki M, Kibata K, Satsutani N, Ogata M, Miyara T, Nomura S (2018). Neutrophil-to-lymphocyte ratio as an early marker of outcomes in patients with advanced non-small-cell lung cancer treated with nivolumab. Int J Clin Oncol.

[16] Triner D, Shah YM (2019). Hypoxic regulation of neutrophils in Cancer. Int J Mol Sci.

[17] Wang X, Qiu L, Li Z, Wang XY, Yi H (2018). Understanding the multifaceted role of neutrophils in Cancer and autoimmune diseases. Front Immunol.

[18] Burnouf T, Goubran HA, Chou ML, Devos D, Radosevic M (2014). Platelet microparticles: detection and assessment of their paradoxical functional roles in disease and regenerative medicine. Blood Rev.

[19] Dovizio M, Bruno A, Contursi A, Grande R, Patrignani P (2018). Platelets and extracellular vesicles in cancer: diagnostic and therapeutic implications. Cancer Metastasis Rev.

[20] Varon D, Hayon Y, Dashevsky O, Shai E (2012). Involvement of platelet derived microparticles in tumor metastasis and tissue regeneration. Thromb Res.

[21] Liu T, Wang J, Liu Y (2021). Prediction of the therapeutic effects of pembrolizumab and nivolumab in advanced non-small cell lung cancer by platelet-derived microparticles in circulating blood. Technol Cancer Res T.

[22] Eisenhauer EA, Therasse P, Bogaerts J, Schwartz LH, Sargent D, Ford R, Dancey J, Arbuck S, Gwyther S, Mooney M, Rubinstein L, Shankar L, Dodd L, Kaplan R, Lacombe D, Verweij J (2009). New response evaluation criteria in solid tumours: revised RECIST guideline (version 1.1). Eur J Cancer.

[23] Seymour L, Bogaerts J, Perrone A (2017). RECIST working group. iRECIST: guidelines for response criteria for use in trials testing immunotherapeutics. Lancet Oncol.

[24] LaPar DJ, Likosky DS, Zhang M, et al. Development of a risk prediction model and clinical risk score for isolated tricuspid valve surgery. Ann Thorac Surg. 2018;106(1):129–36. 10.1016/j.athoracsur.2017.11.077. 10.1016/j.athoracsur.2017.11.077 29410187

[25] Kanazawa S, Nomura S, Kuwana M, Muramatsu M, Yamaguchi K, Fukuhara S (2003). Monocyte-derived microparticles may be a sign of vascular complication in patients with lung cancer. Lung Cancer.

[26] Najjar F, Alammar M, Al-Massarani G (2017). Circulating endothelial cells and microparticles for prediction of tumor progression and outcomes in advanced non-small cell lung cancer. Cancer Biomark.

[27] Mesri M, Altieri DC (1999). Leukocyte microparticles stimulate endothelial cell cytokine release and tissue factor induction in a JNK1 signaling pathway. J Biol Chem.

[28] Mesri M, Altieri DC (1998). Endothelial cell activation by leukocyte microparticles. J Immunol.

[29] Hong Y, Eleftheriou D, Hussain AA (2012). Anti-neutrophil cytoplasmic antibodies stimulate release of neutrophil microparticles. J Am Soc Nephrol.

[30] Anene C, Graham AM, Boyne J, Roberts W (2018). Platelet microparticle delivered microRNA-let-7a promotes the angiogenic switch. Biochim Biophys Acta Mol basis Dis.

[31] Liang H, Yan X, Pan Y (2015). MicroRNA-223 delivered by platelet-derived microvesicles promotes lung cancer cell invasion via targeting tumor suppressor EPB41L3. Mol Cancer.

[32] Helley D, Banu E, Bouziane A, Banu A, Scotte F, Fischer AM, Oudard S (2009). Platelet microparticles: a potential predictive factor of survival in hormone-refractory prostate cancer patients treated with docetaxel-based chemotherapy. Eur Urol.

[33] Kim HK, Song KS, Park YS, Kang YH, Lee YJ, Lee KR, Kim HK, Ryu KW, Bae JM, Kim S (2003). Elevated levels of circulating platelet microparticles, VEGF, IL-6 and RANTES in patients with gastric cancer: possible role of a metastasis predictor. Eur J Cancer.

